# Primary Clear Cell Sarcoma of the Dermis Mimicking Malignant Melanoma

**DOI:** 10.4274/balkanmedj.2017.0796

**Published:** 2018-03-15

**Authors:** Ifeyinwa E. Obiorah, Pauline Brenholz, Metin Özdemirli

**Affiliations:** 1 Department of Pathology, Medstar Georgetown University Hospital, Washington, USA; 2 Department of Medical Genetics, Integrated Oncology, New York, USA

**Keywords:** Clear cell, melanoma, dermis, sarcoma

## Abstract

**Background:**

Clear cell sarcoma is a rare malignant soft tissue neoplasm that typically involves tendons and aponeurosis. Clear cell sarcoma in the dermis is an extremely rare occurrence, and it is difficult to differentiate between this neoplasm and dermal malignant melanoma because they have similar morphologic and immunohistochemical features. Although rare, clear cell sarcoma of the skin typically occurs in the extremities. To our knowledge, there are no reported cases of primary clear cell sarcoma of the skin occurring in the neck. Here, we report an unusual case of clear cell sarcoma arising in the skin of the neck.

**Case Report:**

A 43-year-old female presented with a right neck lesion. Histologic sections of the lesion showed a nodular proliferation of spindle cells with pale cytoplasm with epithelioid features involving the entire dermis with no epidermal component. The tumour cells were positive for melanocytic markers, including S100 and Human Melanoma Black 45, which led to an initial diagnosis of malignant melanoma. Fluorescence *in situ* hybridization showed a rearrangement of the *EWSR1* gene on chromosome 22q12, which led to a diagnosis of primary clear cell sarcoma in the skin.

**Conclusion:**

Because the treatments for clear cell sarcoma and conventional melanoma are different, fluorescence *in situ* hybridization for *EWSR1* should be performed in any dermal lesions with melanocytic features that do not have an *in situ* component.

Clear cell sarcoma was first described by Dr. Franz Enzinger in 1965 as a sarcoma with a distinct morphological pattern arising from the tendons and aponeurosis of extremities (1). The disorder was reclassified by Chung and Enzinger (2) in 1983 as malignant melanoma of the soft parts due to a demonstration of melanin in more than two-thirds of clear cell sarcoma cases. This supports the origin of the tumour from migrated neural crest cells. Clear cell sarcoma of the skin is extremely rare, and few cases have been reported (3,4,5,6). Due to the rare nature of the neoplasm, the tumour can be easily confused with malignant melanoma, since they share similar pathologic and immunohistochemical properties. Although clear cell sarcoma is considered the soft tissue counterpart of malignant melanoma, it can be genetically differentiated from malignant melanoma. A characteristic reciprocal translocation t(12;22) involving the *Ewing’s sarcoma (EWSR1)* gene, which is typically absent in malignant melanoma, is critical for the diagnosis of clear cell sarcoma (7,8). Here we report the clinicopathologic findings of a rare case of primary clear cell sarcoma of the dermis of the neck, in which we integrate morphological and immunohistochemical evaluation with confirmatory molecular studies.

## CASE PRESENTATION

**Figure 1:**
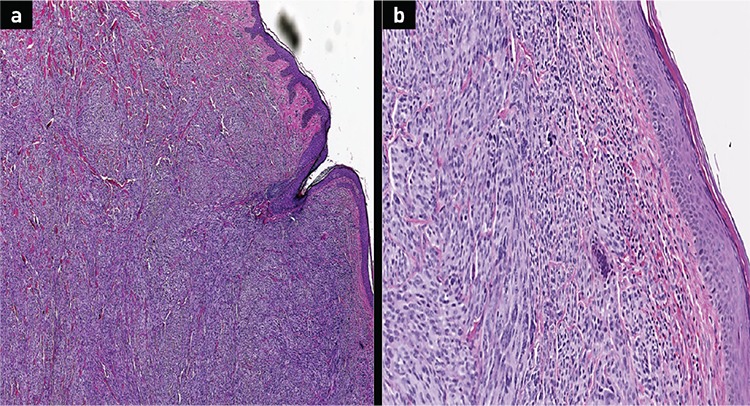
Histopathological examination of the skin. Sections show (a) diffuse infiltration of the dermis with sheets of neoplastic cells (b) without the involvement of the epidermis H&E x40 and x100 magnification respectively.

**Figure 2:**
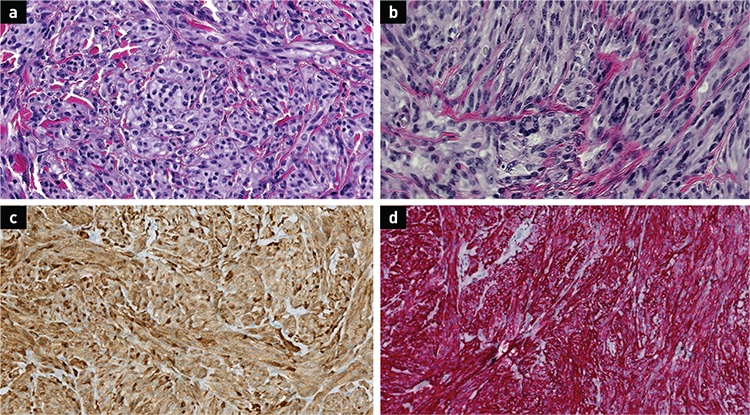
Histologic examination and immunohistochemical staining of the tumour. Higher magnification shows (a) nests and fascicles of spindle cells with clear cytoplasm and (b) wreath-like giant cells H&E x40 magnification. The tumour cells were positive for (c) S-100 and (d) HMB-45 (x400 magnification).

A 43-year-old female presented at a dermatology clinic at an outside institution with a small nodule on the right side of her neck, which had been present for over a year. A biopsy was taken for histopathologic examination, which showed a nodular mass with fascicles of spindle cells (Figure 1) with clear cytoplasm and wreath-like giant cells (Figure 2a, 2b), involving the entire dermis, with focal extension into the superficial subcutaneous tissue.

**Figure 3:**
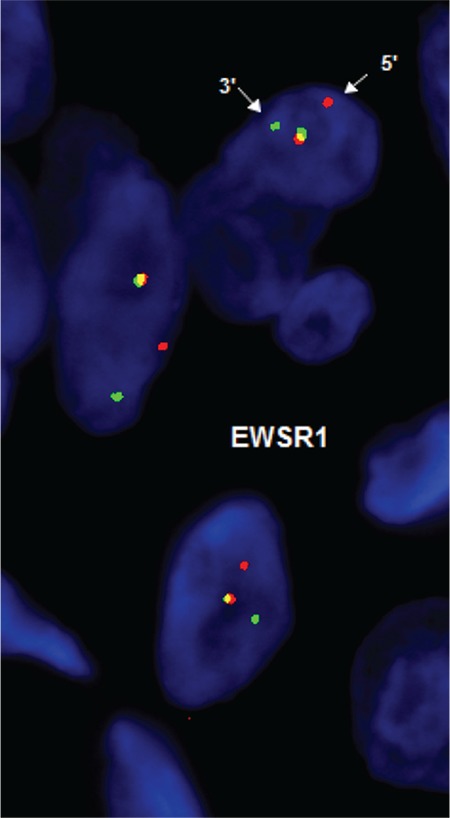
Fluorescence *in situ* hybridization analysis of the mass. The normal pattern consists of a fusion of the orange and green signals, while the rearranged *EWSR1* is seen as a split of the fusion signal into red 5’ centromeric region and green 3’ telomeric region (LSI *EWSR1* Dual Color Break Apart Probe).

Immunohistochemistry was performed on the tissue sections according to the standard method for formalin-fixed paraffin-embedded tissue (FFPET) by the Laboratory Corporation of America Inc. (New Jersey), using the following antibodies; S100, Human Melanoma Black 45 (HMB-45), vimentin, Melan A, factor XIIIa, and CD68. Control stainings were satisfactory. The neoplastic cells were positive for S-100 (Figure 2c), HMB-45 (Figure 2d), and vimentin. The tumour cells were negative for Melan A, factor XIIIa, and CD68. The results led to a diagnosis of malignant melanoma of the dermis (Clarke Level V). Because of the unusual histologic presentation, which included the presence of clear cells, multinucleated giant cells, and with no epidermal *in situ* component, the differential diagnosis included clear cell sarcoma and Spitz nevus. The lesion was re-excised, and sections of the re-excision were negative for any residual tumour. The case was sent out for an expert consultation to two facilities, which agreed with the initial diagnosis of malignant melanoma but could not exclude intradermal clear cell sarcoma. Fluorescence *in situ* hybridization (FISH) was ordered on the specimen, and the case was sent to our institution for another opinion. Upon examination, there was a nodular proliferation of malignant spindle cells with clear cytoplasm and wreath-like giant cells, occasional mitotic figures, and no obvious melanin. The tumour measured 0.5 cm and involved the dermis and the subcutaneous tissue, with no epidermal involvement. FISH was performed using the standard method for FFPET with the Abbott Molecular LSI *EWSR1* Dual Color Break Apart Probe (Illinois, United States). According to protocol 200, interphase cells were examined, and 59.5% of these were positive for the split signals, indicating a positive rearrangement of the *EWSR1* gene at chromosome 22q12 (Figure 3). Based on the FISH results and the lack of an *in situ* involvement, the diagnosis of a dermal clear cell sarcoma was made, rather than nodular malignant melanoma. Physical and imaging studies showed no evidence of metastasis. The patient was initially scheduled for prophylactic IL-2 treatment for melanoma. However, once the diagnosis was changed to primary clear cell sarcoma, no further treatment was initiated. The patient is currently disease free, with no evidence of metastatic disease 6 months after initial biopsy. An Institutional Review Board waiver of consent was obtained for the case report.

## DISCUSSION

Clear cell sarcoma is a rare, aggressive malignant neoplasm, accounting for 1% of all soft tissue sarcomas. This type of tumour frequently affects tendons and aponeurosis of the limbs (1,9) and the highest incidence is found between the ages of 20 and 40 years (9). Histologically, the tumours consist of nests or fascicles of fusiform or epithelioid cells with a clear to granular eosinophilic cytoplasm, vesicular nuclei with prominent nucleoli, and occasional multinucleated giant cells (2,10). Clinical characteristics include an invasive growth pattern, tendency for recurrence, lymphatic spread, and lung metastases (2,8). Poor prognosis is associated with tumour size greater than 5 cm, the presence of necrosis, metastasis, and local recurrence (11). Due to the histomorphologic overlap, clear cell sarcoma can be easily misdiagnosed as malignant melanoma. Molecular analysis remains the key to diagnosis. Studies have shown that clear cell sarcoma has the reciprocal translocation t(12;22)(q13;q12), which has not yet been identified in malignant melanoma (7,8). The gene fusion product involves EWSR (22q12) and activating transcription factor (*12q13*) genes, and the translocation has been detected in about 75% of reported cases of clear cell sarcoma (7,8). Another translocation of EWSR to CREB1, a gene at 2q13, has also been described in a subset of clear cell sarcomas (4,12). Clear cell sarcomas of the skin, although rare, have been reported.

A review of all reported 23 cases of clear cell sarcoma of the skin (including our case) (Table 1) showed that the mean age of the patients was 30.65±19.62 and they were predominantly female (65.21%). The tumours ranged in size from 0.4 to 3 cm (mean: 1.1±0.6 cm) and tumour size was unknown in three patients. The majority of the cases occurred in the extremities (78.26%). In all cases, the tumour cells were present in the dermis, arranged in nests and fascicles, separated by fibrous septa. Ten cases showed that the nests bordered but did not involve the epidermis, thus resembling junctional nests of a melanocytic tumour. There was a lack of involvement of epidermis in 22 cases; only one case had transepidermal spread. Six cases showed focal involvement with the subcutaneous tissue. The cells showed necrosis in 13% of cases, and the number of mitoses was less than 10 per high power fields (HPF) in 18 patients (78.3%). HMB-45 was positive in 17 tumours (73.91%), while S-100 protein was largely positive in 22 patients (95.65%). Melan A and microphthalmia transcription factor were expressed in 52.17% and 4.34% of cases, respectively. The EWS/ATF-1 fusion transcript was detected in 22 patients; only one patient had the EWSR/CREB1 rearrangement. Thirteen patients (56.52%) presented with localized disease and all of them underwent surgical resection of the primary tumour. Of the 13 patients, re-excision was done on five patients with positive surgical margins and one patient received adjuvant radiotherapy. One patient had a local recurrence, which was re-excised. Three patients (13.04%) presented with metastatic disease to lymph nodes. Three patients had metastatic disease to both lymph nodes and other organs, including bone. These patients were treated with surgery. Only one patient with extensive metastatic disease received surgery, chemotherapy, and radiotherapy. Fourteen patients (60.87%) with localized disease, including a local recurrence, were alive with no evidence of the disease and a mean follow up of 27±20.16 months. Of the six patients who had metastatic disease, two (8.7%) died of the tumours, two are alive with disease, one was lost during follow-up, and the outcome was unknown in another patient.

Similar to the majority of cases of clear cell sarcoma, cutaneous clear cell sarcomas tend to occur in the extremities. Although cutaneous clear cell sarcoma has been reported in the head region (13,14), to our knowledge, here we report for the first time the identification of primary clear cell sarcoma of the dermal skin in the neck. Based on our review, most cases of clear cell sarcoma of the skin were associated with good prognostic features, which included small tumour size, lack of necrosis, and decreased rate of local recurrence and metastasis. Treatment with wide local excision as soon as the diagnosis is established should remain the mainstay of treatment. Typically, chemotherapy and radiotherapy have not been shown to be beneficial in clear cell sarcoma, especially in patients with metastatic disease (9). Therefore, it is important to diagnose this entity early and distinguish it from conventional malignant melanoma since further treatments differ significantly.

In conclusion, the rare diagnosis of cutaneous clear cell sarcoma can be made using the stated morphologic and immunohistochemical features, but it is important to differentiate this tumour from malignant melanoma. Molecular confirmation of the sarcoma using FISH studies can lead to early diagnosis and treatment, which can improve the outcome of the disorder.

## Figures and Tables

**Table 1:**
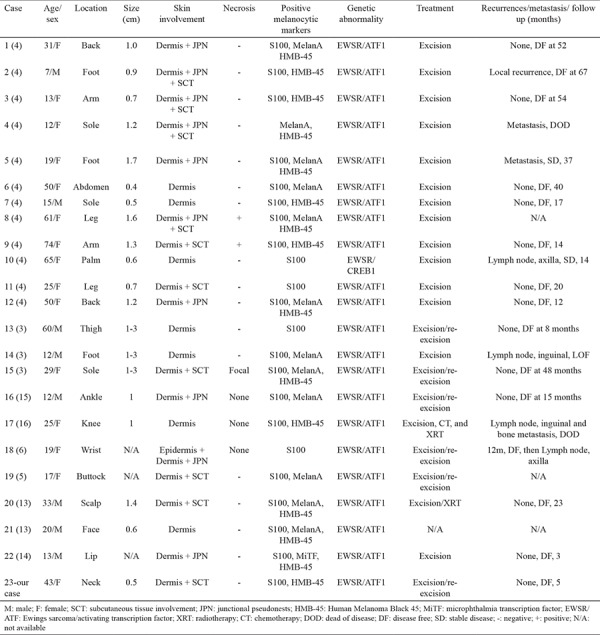
Reported cases of cutaneous clear cell sarcoma
